# QSAR Analysis of Lichen Depsides and Derivatives: Electronic Descriptors as Predictors of Antioxidant Activity via PLS-1

**DOI:** 10.3390/antiox15050584

**Published:** 2026-05-05

**Authors:** Patricia Mollinedo, José Luis Vila, Paola Nogales-Ascarrunz, Luis Apaza Ticona

**Affiliations:** 1 Chemistry Research Institute, Faculty of Pure and Natural Sciences, Universidad Mayor de San Andrés, Building, 2nd Floor, Laboratory 6, Calle 27, Cota Cota, University Campus, La Paz 10077, Bolivia; pmollinedo@fcpn.edu.bo (P.M.); jlvila@fcpn.edu.bo (J.L.V.); pnogalesa@fcpn.edu.bo (P.N.-A.); 2 Organic Chemistry Unit, Department of Chemistry in Pharmaceutical Sciences, Faculty of Pharmacy, University Complutense of Madrid, Plza. Ramón y Cajal s/n, 28040 Madrid, Spain

**Keywords:** lichen-derived depsides, QSAR, electronic descriptors, phenoxyl radical, PLS-1, TEAC assay

## Abstract

The antioxidant activity of natural and semi-synthetic depsides and benzofurans—including *R*-(+)-usnic acid (**1**), dibenzoyl usnic acid (**2**), atranorin (**3**), 2,4-bis(benzoyloxy)atranorin (**4**), 4-*O*-methyl atranorin (**5**), decarboxythamnolic acid (**6**), thamnolic acid (**7**), and perlatolic acid (**8**)—was evaluated in this study. Natural compounds were isolated from selected lichen species, whilst semi-synthetic derivatives were prepared to investigate the influence of esterification and methylation on electronic properties and radical-scavenging capacity. Structural elucidation was performed using NMR spectroscopy and mass spectrometry (MS). Electronic and thermodynamic descriptors, including the bond dissociation energy (BDE) of the most reactive O–H group, HOMO and LUMO_r_ energies, the HOMO–HOMO-1 energy gap (ΔH(H-1)), polarisability, and logP, were calculated and correlated with experimentally determined antioxidant capacity using the TEAC assay. Multivariate partial least squares (PLS-1) analysis identified ΔH(H-1) and LUMO_r_ as the primary determinants of antioxidant activity, with BDE and ΔHf providing complementary contributions. Perlatolic acid (**8**) exhibited the highest radical-scavenging capacity (TEAC = 2.7), whereas *R*-(+)-usnic acid (**1**) and dibenzoyl usnic acid (**2**) were the least active compounds (TEAC ≈ 0.1). Antioxidant activity was found not to correlate with the number of hydroxyl groups, but rather to be governed by electronic redistribution, conjugation effects, and substituent modulation. Exclusion of the outlier decarboxythamnolic acid (**6**) improved model performance, explaining 79.8% of the variance in TEAC values (R^2^Y), with strong predictive ability (Q^2^ = 0.724) using a single latent variable. Overall, these findings provide a robust basis for the prediction and rational design of new antioxidant compounds, highlighting the relevance of lichen-derived metabolites as structurally stable scaffolds with potential applications in pharmaceutical and nutraceutical development.

## 1. Introduction

Oxidative stress is a fundamental biochemical process in human physiology, arising from an imbalance between the production of reactive oxygen species (ROS) and the organism’s antioxidant defences [1]. ROS comprise both free radicals, such as superoxide (O_2_•^−^) and hydroxyl radicals (HO•), and non-radical species, including hydrogen peroxide (H_2_O_2_) and singlet oxygen (^1^O_2_). These species differ in reactivity and half-life and selectively affect lipids, proteins, and nucleic acids [2]. Owing to their high reactivity, ROS readily interact with key cellular components, leading to lipid peroxidation, protein modification, and DNA damage [3]. The accumulation of ROS, together with disruption of the redox balance within endogenous antioxidant defence systems, has been associated with numerous pathological conditions, including cardiovascular diseases, neurodegenerative disorders such as Alzheimer’s and Parkinson’s diseases, diabetes, cataracts, and various forms of cancer [4]. Consequently, the identification of antioxidant agents capable of neutralising ROS and protecting biological systems from oxidative damage remains a major focus of research [5].

To counteract oxidative stress, antioxidant systems have evolved as protective mechanisms that neutralise free radicals and prevent irreversible cellular damage [6]. These systems can be broadly classified as endogenous or exogenous. Endogenous antioxidant defences include enzymatic components such as catalase (CAT), glutathione peroxidase (GPx), and superoxide dismutase (SOD), together with non-enzymatic constituents, including reduced glutathione, bilirubin, and metal-binding proteins that regulate free radical generation mediated by metal ions [7]. Exogenous antioxidants comprise vitamins (A, C, and E), minerals (Cu, Mn, Se, and Zn), carotenoids, and polyphenols present in plant-derived foods, as well as synthetic compounds used in food, cosmetic, and pharmaceutical formulations [8]. These compounds act through several mechanisms, including radical scavenging via hydrogen or electron donation (type I), inhibition of ROS formation through metal chelation or decomposition of reactive species (type II), and physical protection mechanisms, such as shielding against radiation or stabilisation under controlled conditions (type III) [9]. In this context, natural antioxidants have attracted increasing attention owing to their structural diversity and their potential to complement endogenous defence systems against oxidative stress [10].

Among natural sources, lichens are particularly noteworthy for their structurally diverse secondary metabolites, which exhibit a wide range of biological activities. Lichens inhabiting extreme environments often develop adaptive biochemical strategies involving the production of phenolic compounds capable of modulating oxidative processes [11]. Many of these compounds contain phenolic hydroxyl groups that play a crucial role in antioxidant activity by facilitating hydrogen atom donation and stabilising phenoxyl radicals. The Bolivian flora, characterised by remarkable biodiversity and unique ecological niches, provides favourable conditions for the biosynthesis of such specialised compounds [12,13]. In high-altitude regions such as the Altiplano, organisms are exposed to intense ultraviolet radiation and arid conditions, which promote oxidative stress and favour the production of protective secondary metabolites [14,15]. As part of these adaptive responses, lichens synthesise compounds capable of neutralising ROS and limiting lipid peroxidation [16]. This protective function not only contributes to lichen survival but also highlights the pharmacological potential of these compounds in preventing human diseases associated with oxidative stress, including cataracts, diabetes, vascular disorders, cancer, and neurodegenerative diseases [17]. Accordingly, these natural products represent promising candidates for pharmacological, nutraceutical, and cosmetic applications [18].

Lichen-derived secondary metabolites, particularly depsides and benzofuran derivatives, have attracted considerable attention owing to their diverse biological properties. Compounds such as usnic acid exhibit both antioxidant and photoprotective activities through efficient ultraviolet absorption and ROS scavenging mechanisms [19,20]. The antioxidant capacity of these phenolic compounds is strongly influenced by structural factors, including the number and position of hydroxyl groups, the degree of conjugation within the aromatic system, and the stability of the phenoxyl radicals formed upon hydrogen atom donation [21]. Conversely, substituents such as glycosides or strongly electron-withdrawing groups may reduce antioxidant efficiency through steric and electronic effects that alter electron density distribution [22]. These structural features are therefore critical in determining the biological activity of phenolic compounds.

Although several depside and benzofuran derivatives exhibit notable antioxidant and photoprotective properties, the contribution of specific electronic parameters governing their radical scavenging capacity remains insufficiently explored. In particular, the role of phenolic hydroxyl groups and their influence on electronic descriptors associated with antioxidant activity have not been fully elucidated, highlighting the need for integrated experimental and computational studies [23,24].

Computational approaches are now widely used to explore antioxidant mechanisms and to predict structure–activity relationships (SARs). Relevant descriptors of antioxidant capacity include parameters such as formation energies, bond dissociation energies (BDE), and frontier molecular orbitals, namely the highest occupied molecular orbital (HOMO) and the lowest unoccupied molecular orbital (LUMO) of the resulting radical species [25]. These electronic descriptors provide insight into the ability of phenolic compounds to donate hydrogen atoms or electrons, processes that are fundamental to radical scavenging reactions.

In the present study, we focus on the isolation of depside derivatives from lichens and the preparation of strategically designed semisynthetic benzofuran derivatives to evaluate the role of phenolic hydroxyl groups in antioxidant activity. These semisynthetic modifications were undertaken to investigate how structural changes affecting these functional groups influence electronic properties and radical stabilisation capacity. To rationalise the experimental findings, electronic descriptors obtained from molecular mechanics, semi-empirical methods, and quantum chemical calculations were analysed, including formation energy, BDE, HOMO, LUMO, polarisability, and lipophilicity. This integrated experimental–computational approach aims to elucidate structure–activity relationships in phenolic antioxidants derived from lichens, thereby contributing to the rational design of bioactive compounds for pharmacological and cosmetic applications.

## 2. Materials and Methods

### 2.1. General Experimental Procedures

High-quality analytical-grade solvents (Merck) were used for the extraction of compounds from *Alectoria ochroleuca* (Schrank) A. Massal., *Everniastrum cirrhatum* (E. Fr.) Hale ex Sipman, *Thamnolia vermicularis* (Sw.) Ach. ex Schaer, and *Stereocaulon ramulosum* Raeusch. Thin-layer chromatography (TLC) was carried out on silica gel plates (Silica gel 60 GF_254_, Merck, Cat. No. 112926-00-8, St. Louis, MO, USA). Spots were visualised using phosphomolybdic acid solution (≥99.99%, Merck, Cat. No. 51429-74-4, St. Louis, MO, USA) and UV light (Spectroline^®^ E-Series UV lamp, 254 nm, 230 V, New York, NY, USA).

Silica gel column chromatography (40–63 µm, SiO_2_, Merck, Cat. No. 112926-00-8, St. Louis, MO, USA) was used for purification, employing solvent systems as detailed in the extraction and isolation section. NMR spectra were recorded on a Bruker Avance DRX 400 spectrometer at 400 MHz (^1^H) and 100 MHz (^13^C). Deuterated chloroform (CDCl_3_, 99.8 atom % D, Merck, Cat. No. 865-49-6, Darmstadt, Germany) and deuterated dimethyl sulfoxide ((CD_3_)_2_SO, 99.8 atom % D, Merck, Cat. No. 2206-27-1, Darmstadt, Germany) were used as solvents.

Fast atom bombardment mass spectrometry (FAB-MS) analyses were performed on a Bruker MAXIS II instrument in positive ion mode ([M + H]^+^). Samples (1–2 mg) were dissolved in 3-nitrobenzyl alcohol (≥99%, Merck, Cat. No. 93-35-6, St. Louis, MO, USA), used as matrix, and introduced directly into the ion source via a sample probe. The operating conditions were as follows: end plate voltage 500 V, capillary voltage 3500 V, nebuliser pressure 0.2 bar, dry gas flow 2.0 L/min, and drying temperature 250 °C, with a mass range of 50–3000 Da. All analyses were performed immediately after sample preparation to minimise matrix degradation.

### 2.2. Extraction and Isolation

Lichen specimens were collected in the Pongo community near La Paz, Bolivia (3500–4000 m a.s.l.; 16°19′42.6″ S, 67°57′17.6″ W). Taxonomic identification was performed at the Herbario Nacional de Bolivia under the supervision of Prof. José Luis Vila, and voucher specimens were deposited as follows: *A. ochroleuca* (000145/Jul/2005), *E. cirrhatum* (000241/Oct/2004), *T. vermicularis* (002019/Sep/2004), and *S. ramulosum* (000336/Nov/2004). Plant material was cleaned, air-dried at room temperature (25 ± 5 °C) for 48 h, and ground into a fine powder prior to extraction.

*A. ochroleuca* (56.0 g) was extracted with acetone (Me_2_CO, ≥99.5%, Merck, Cat. No. 67-64-1, St. Louis, MO, USA; 500 mL) at room temperature (25 ± 5 °C) over 72 h. During the extraction, yellow crystals of *R*-(+)-usnic acid (**1**) formed and were subsequently concentrated *in vacuo*. The resulting crystals were recrystallised from dichloromethane (DCM, ≥99.5%, Merck, Cat. No. 75-09-2, St. Louis, MO, USA; 300 mL) and precipitated upon addition of methanol (MeOH, 99.8%, Merck, Cat. No. 67-56-1, St. Louis, MO, USA; 200 mL), affording 5.1 g (9.1% of dry material).

Compound **1** was then subjected to benzoylation with benzoyl chloride (BzCl, ≥99%, Merck, Cat. No. 98-88-4, St. Louis, MO, USA; 0.5 mL) in chloroform (CHCl_3_, ≥99.5%, Merck, Cat. No. 67-66-3, St. Louis, MO, USA; 20 mL), using pyridine (Py, ≥99%, Merck, Cat. No. 110-86-1, St. Louis, MO, USA; 5 mL) as base. The reaction mixture was stirred for 24 h at 18 °C, yielding dibenzoyl usnic acid (**2**).

*E. cirrhatum* (156.0 g) was extracted with acetone (Me_2_CO, 1 L, ≥99.5%, Merck, Cat. No. 67-64-1, St. Louis, MO, USA) at room temperature (25 ± 5 °C) for 72 h. The crude extract (21.6 g) was concentrated *in vacuo* and washed successively three times with dichloromethane (DCM, 500 mL × 3, ≥99.5%, Merck, Cat. No. 75-09-2, St. Louis, MO, USA) and ethyl acetate (EtOAc, 500 mL × 3, 99.8%, Merck, Cat. No. 141-78-6, St. Louis, MO, USA). The soluble fraction was then concentrated under reduced pressure and subjected to silica gel column chromatography, affording atranorin (**3**, 9.0 g) as a white solid, which was recrystallised from EtOAc (300 mL).

From this material, 6.0 g of atranorin (**3**, 16.1 mmol) were dissolved in chloroform (40 mL, CHCl_3_, ≥99.0–99.5%, Merck, Cat. No. 67-66-3, St. Louis, MO, USA) together with pyridine (3.9 mL, ≥99.5%, Merck, Cat. No. 110-86-1, St. Louis, MO, USA). Benzoyl chloride (4.7 mL, BzCl, ≥99%, Merck, Cat. No. 98-88-4, St. Louis, MO, USA) was then added dropwise at 18 °C. After stirring for 24 h, standard work-up afforded 2,4-bis(benzoyloxy)atranorin (**4**, 8.4 g, 85%) as a white solid.

Subsequently, 4.2 g of compound **4** were selectively deprotected at the 4-position and methylated with iodomethane (0.5 mL, CH_3_I, 99%, Merck, Cat. No. 74-88-4, St. Louis, MO, USA) in dimethylformamide (DMF, 20 mL, ≥99%, Merck, Cat. No. 68-12-2, St. Louis, MO, USA) at room temperature (25 ± 5 °C) over 12 h. Purification by silica gel chromatography (*n*-hexane/EtOAc, 8:2 → 6:4) afforded 4-*O*-methyl atranorin (**5**) as a white solid.

*T. vermicularis* (20.0 g) was extracted with acetone (Me_2_CO, 1 L, ≥99.5%, Merck, Cat. No. 67-64-1, St. Louis, MO, USA; 200 mL) at room temperature (25 ± 5 °C) for 72 h. The crude extract (1.4 g) was concentrated *in vacuo* and washed at least five times with acetone (200 mL each). The resulting material was subjected to silica gel column chromatography using dichloromethane/acetone mixtures of increasing polarity, yielding the meta-depsides decarboxythamnolic acid (**6**, 89 mg) and thamnolic acid (**7**, 1.2 g).

*S. ramulosum* (254.0 g) was sequentially extracted by maceration at room temperature (25 ± 5 °C) for 72 h with petroleum ether (PE, 1 L, 99.5%, Merck, Cat. No. 8012-95-1, St. Louis, MO, USA), dichloromethane (DCM, 1 L, ≥99.5%), acetone (Me_2_CO, 1 L, ≥99.5%), and ethanol (EtOH, 1 L, 99.5%, Merck, Cat. No. 64-17-5, St. Louis, MO, USA). The combined extracts were filtered and concentrated under reduced pressure to yield 14.6 g of crude extract. This was further fractionated by silica gel chromatography using DCM/MeOH mixtures of increasing polarity, affording guanine (1, 40 mg), perlatolic acid (**8**, 250 mg), methyl *β*-orcinolcarboxylate (65 mg), atranorin (800 mg), and galactitol (32 mg).

### 2.3. Spectroscopic Data

#### 2.3.1. *R*-(+)-Usnic Acid (**1**)

Characteristics: yellow crystals; m.p. 201–202 °C (CHCl_3_); ^1^H NMR (400 MHz, CDCl_3_) *δ_H_*: 11.10 (s, 1H, OH-9), 5.98 (s, 1H, H-4), 2.68 (s, 3H, CH_3_CO-6), 2.67 (s, 3H, CH_3_CO-2), 2.14 (s, 3H, CH_3_-8), 1.77 (s, 3H, CH_3_-9b); ^13^C NMR (100 MHz, CDCl_3_) *δ_C_*: 202.2 (CO-2), 200.8 (CO-6), 198.5 (C-1), 179.8 (C-4a), 164.3 (C-7), 157.9 (C-9), 152.4 (C-9a), 147.1 (C-3), 109.7 (C-8), 105.7 (C-4a), 101.9 (C-6a), 100.9 (C-5a), 59.5 (C-9b), 29.4 (CH_3_CO-6), 29.2 (CH_3_CO-2), 19.9 (CH_3_-8), 8.7 (CH_3_-9b); FABMS *m/z* 345 [M + H]^+^ (C_18_H_16_O_7_). Data were compared with literature values [26,27].

#### 2.3.2. Dibenzoyl Usnic Acid (**2**)

Characteristics: yellow solid; m.p. 189–191 °C (CH_2_Cl_2_–EtOAc); ^1^H NMR (400 MHz, CDCl_3_) *δ_H_*: 10.41 (s, 1H, OH-9), 7.91 (d, 2H, Ar–H, benzoyl), 7.72 (d, 2H, Ar–H, benzoyl), 7.00–8.00 (m, 4H, Ar–H, benzoyl), 5.92 (s, 1H, H-4), 5.45 (d, *J* = 1.76 Hz, 1H, H-12b), 5.12 (d, *J* = 1.76 Hz, 1H, H-12a), 2.49 (s, 3H, CH_3_CO-6), 1.96 (s, 3H, CH_3_-8), 1.74 (s, 3H, CH_3_-9b); ^13^C NMR (100 MHz, CDCl_3_) *δ_C_*: 201.2 (C-1), 200.9 (CO-6), 174.2 (C-4a), 165.4 (C-3), 164.7 (CO–Ph), 164.2 (C-7), 163.3 (CO–Ph), 157.8 (C-9), 135.0 (C-12), 133.9 (C_ipso_–Ph), 130.1 (CH–Ph), 128.7 (CH–Ph), 114.9 (C-2), 110.2 (CH_2_-12), 109.3 (C-8), 104.3 (C-9a), 102.1 (C-6), 61.0 (C-9b); FABMS *m/z* 569 [M + H]^+^ (C_32_H_24_O_9_). Data were compared with literature values [28].

#### 2.3.3. Atranorin (**3**)

Characteristics: white crystals; m.p. 196–197 °C (CH_2_Cl_2_–EtOAc); ^1^H NMR (400 MHz, CDCl_3_) *δ_H_*: 12.59 (s, 1H, OH-4), 12.53 (s, 1H, OH-2), 11.95 (s, 1H, OH-2′), 10.39 (s, 1H, H-8), 6.56 (s, 1H, H-5′), 6.44 (s, 1H, H-5), 4.05 (s, 3H, OCH_3_-7′), 2.71 (s, 3H, H-9), 2.59 (s, 3H, H-9′), 2.11 (s, 3H, H-8′); ^13^C NMR (100 MHz, CDCl_3_) *δ_C_*: 172.4 (C-7′), 169.9 (C-7), 169.3 (C-2), 163.1 (C-2′), 161.2 (C-4), 152.6 (C-6), 152.2 (C-4′), 140.1 (C-6′), 117.0 (C-3′), 116.2 (C-5′), 113.1 (C-5), 110.5 (C-1′), 108.8 (C-3), 103.1 (C-1), 52.1 (OCH_3_), 25.6 (C-9), 24.2 (C-8′), 20.8 (C-8), 19.2 (C-9′); FABMS *m/z* 375 [M + H]^+^ (C_19_H_18_O_8_). Data were compared with literature values [29].

#### 2.3.4. 2,4-bis(benzoyloxy)atranorin (**4**)

Characteristics: white solid; m.p. 215–217 °C (CH_2_Cl_2_–EtOAc); ^1^H NMR (400 MHz, CDCl_3_) *δ_H_*: 11.95 (s, 1H, OH-2′), 10.29 (s, 1H, H-8), 8.25 (m, 4H, Ar–H *ortho*), 7.75 (m, 2H, Ar–H *para*), 7.55 (m, 4H, Ar–H *meta*), 7.30 (s, 1H, H-5), 6.20 (s, 1H, H-5′), 3.95 (s, 3H, OCH_3_-7′), 2.69 (s, 3H, H-9), 2.31 (s, 3H, H-8′), 2.05 (s, 3H, H-9′); ^13^C NMR (100 MHz, CDCl_3_) *δ_C_*: 172.4 (C-7′), 165.3 (C=O–Ph), 164.8 (C=O–Ph), 152.6 (C-6), 152.2 (C-4′), 140.5 (C-1), 140.1 (C-6′), 133.9 (C_ipso_–Ph), 130.1 (CH–Ph), 128.7 (CH–Ph), 123.3 (C-3′), 116.2 (C-5′), 113.1 (C-5), 109.7 (C-3), 103.1 (C-1), 52.3 (OCH_3_), 25.6 (C-9), 24.2 (C-8′), 20.5 (C-8), 19.9 (C-9′); FABMS *m/z* 503 [M + H]^+^ (C_27_H_22_O_10_). Data were compared with literature values [30].

#### 2.3.5. 4-*O*-methyl Atranorin (**5**)

Characteristics: white solid; m.p. 198–200 °C (CH_2_Cl_2_–EtOAc); ^1^H NMR (400 MHz, CDCl_3_) *δ_H_*: 12.58 (s, 1H, OH-2), 11.92 (s, 1H, OH-2′), 10.30 (s, 1H, H-8), 6.59 (s, 1H, H-5), 6.32 (s, 1H, H-5′), 3.98 (s, 3H, OCH_3_-4), 3.96 (s, 3H, OCH_3_-7′), 2.55 (s, 3H, H-9′), 2.53 (s, 3H, H-9), 2.11 (s, 3H, H-8′); ^13^C NMR (100 MHz, CDCl_3_) *δ_C_*: 172.8 (C-7′), 169.8 (C-7), 165.2 (C-4), 163.3 (C-2′), 162.1 (C-2), 153.6 (C-4′), 150.1 (C-6), 140.0 (C-6′), 117.5 (C-3′), 116.8 (C-5′), 114.2 (C-1), 110.2 (C-1′), 109.2 (C-3), 104.2 (C-5), 52.4 (OCH_3_-4), 52.1 (OCH_3_-7′), 25.3 (C-9), 24.1 (C-8′), 22.3 (C-8), 19.9 (C-9′); FABMS *m/z* 389 [M + H]^+^ (C_20_H_20_O_8_). Data were compared with literature values [30].

#### 2.3.6. Decarboxythamnolic Acid (**6**)

Characteristics: white solid; m.p. 216 °C (CH_2_Cl_2_–EtOAc); ^1^H NMR (400 MHz, DMSO-*d_6_*) *δ_H_*: 12.45 (s, 1H, OH-2), 10.23 (s, 1H, H-8′), 8.95 (s, 1H, OH-2′), 6.65 (s, 1H, H-5), 6.38 (s, 1H, H-5′), 3.90 (s, 3H, OCH_3_-4), 2.50 (s, 3H, H-8), 2.28 (s, 3H, H-3′); ^13^C NMR (100 MHz, DMSO-*d_6_*) *δ_C_*: 169.5 (C-7), 165.8 (C-4), 162.3 (C-2′), 161.5 (C-2), 154.0 (C-4′), 145.1 (C-6), 140.8 (C-6′), 112.4 (C-1), 109.8 (C-3′), 106.3 (C-5), 104.2 (C-1′), 102.1 (C-3), 52.1 (OCH_3_-4), 25.4 (C-8), 22.3 (C-8′), 17.9 (C-7′); FABMS *m/z* 377 [M + H]^+^ (C_18_H_16_O_9_). Data were compared with literature values [31].

#### 2.3.7. Thamnolic Acid (**7**)

Characteristics: white solid; m.p. 224 °C (CH_2_Cl_2_–EtOAc); ^1^H NMR (400 MHz, DMSO-*d_6_*) *δ_H_*: 10.40 (s, 1H, CHO-8′), 6.15 (s, 1H, H-5), 6.13 (s, 1H, H-5′), 3.86 (s, 3H, OCH_3_-4), 2.50 (s, 3H, CH_3_-8), 2.45 (s, 3H, CH_3_-7′); ^13^C NMR (100 MHz, DMSO-*d_6_*) *δ_C_*: 194.9 (CHO-8′), 172.5 (C-7), 166.8 (C-2′), 164.5 (C-9), 161.7 (C-4), 161.0 (C-2), 157.3 (C-4′), 145.4 (C-6), 143.9 (C-6′), 129.9 (C-5′), 112.5 (C-1), 110.2 (C-1′), 109.5 (C-3′), 106.5 (C-5), 105.5 (C-3), 55.6 (OCH_3_-4), 22.3 (C-8), 16.4 (C-7′); FABMS *m/z* 421 [M + H]^+^ (C_19_H_16_O_11_). Data were compared with literature values [31].

#### 2.3.8. Perlatolic Acid (**8**)

Characteristics: white crystals; m.p. 192–194 °C (CH_2_Cl_2_–EtOAc); ^1^H NMR (400 MHz, CDCl_3_) *δ_H_*: 6.72 (d, *J* = 1.7 Hz, 1H, H-5′), 6.63 (d, *J* = 1.7 Hz, 1H, H-3′), 6.39 (br s, 2H, H-3, H-5), 3.85 (s, 3H, OCH_3_-4′), 2.96 (t, 2H, H-1a), 2.96 (t, 2H, H-1b), 2.65 (m, 2H, H-2a), 2.65 (m, 2H, H-2b), 2.40 (m, 2H, H-3a), 2.40 (m, 2H, H-4a), 2.25 (m, 2H, H-3b), 2.25 (m, 2H, H-4b), 0.93 (t, 3H, H-5a), 0.87 (t, 3H, H-5b); ^13^C NMR (100 MHz, CDCl_3_) *δ_C_*: 175.8 (C-7), 169.8 (C-9), 166.5 (C-2), 165.3 (C-4), 155.5 (C-4′), 150.5 (C-6′), 148.9 (C-6), 116.7 (C-5′), 111.9 (C-5), 109.4 (C-1′), 104.0 (C-1), 99.4 (C-3), 55.8 (OCH_3_-4′), 37.7 (C-1a), 29.6 (C-1b), 32.5 (C-2a), 32.4 (C-2b), 32.3 (C-3a), 31.8 (C-3b), 23.0 (C-4a), 22.9 (C-4b), 14.1 (C-5a), 13.9 (C-5b); FABMS *m/z* 445 [M + H]^+^ (C_25_H_32_O_7_). Data were compared with literature values [32,33].

### 2.4. Computational Chemistry

Molecular modelling was performed using Spartan’18 (Wavefunction Inc., Irvine, CA, USA). Geometry optimisations were carried out using the semi-empirical AM1 method with a Polak–Ribiere conjugate gradient algorithm under an unrestricted Hartree–Fock (UHF) formalism for open-shell systems. Convergence criteria were set at 0.01 kcal·mol^−1^, with an RMS gradient threshold of 0.1 kcal·Å^−1^·mol^−1^. All calculations were performed *in vacuo*, without inclusion of solvent effects. This method was selected based on its balance between computational efficiency and suitability for comparative analysis of electronic descriptors across structurally related compounds.

### 2.5. Antioxidant Activity Assay

Antioxidant activity was evaluated using the ABTS radical cation decolourisation assay (ABTS•^+^), with Trolox employed as the reference standard. Results were expressed as Trolox Equivalent Antioxidant Capacity (TEAC). The ABTS chromophore (diammonium salt; Merck, Cat. No. 30931-67-0, St. Louis, MO, USA) was dissolved in PBS (7 mM) and oxidised with potassium persulfate (2.45 mM) for 16 h at room temperature in the dark, generating the ABTS•^+^ radical cation (λ_max = 734 nm), which exhibits a blue-to-green colour transition upon reduction. Absorbance was measured at 734 nm using a microplate reader (µQuant Biotek plate reader) after incubation of samples for 30 min at room temperature (25 ± 5 °C) under dark conditions. The assay quantifies radical scavenging activity via a dose-dependent decrease in ABTS•^+^ absorbance.

TEAC values (mmol Trolox equivalents per gram) were calculated from a calibration curve constructed using Trolox (6-hydroxy-2,5,7,8-tetramethylchroman-2-carboxylic acid; Merck, Cat. No. 53188-07-1, St. Louis, MO, USA; 0–250 μM) as the reference standard. Antioxidant activity was classified as high (TEAC ≈ 5, comparable to quercetin), moderate (TEAC 1–5), or weak (TEAC ≤ 1, Trolox equivalent) [34].

### 2.6. Statistical Analysis

Relationships between calculated molecular descriptors and experimental antioxidant activity (TEAC) values were analysed using partial least squares regression (PLS-1), implemented in R (v4.4.2, R Core Team, 2024; Vienna, Austria) with the pls package. All variables were mean-centred and scaled to unit variance prior to modelling. The optimal number of latent variables was determined by minimisation of prediction error using leave-one-out cross-validation (LOOCV). Model performance was assessed using the cross-validated root mean square error of prediction (RMSEP) and the coefficient of determination (r^2^) between predicted and experimentally observed TEAC values.

## 3. Results

### 3.1. Isolation and Characterisation of Compounds

**Figure 1:**
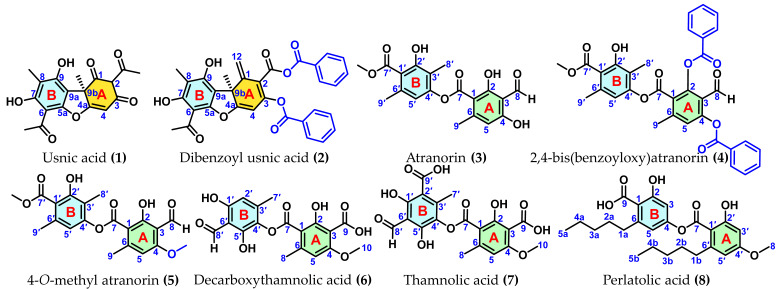
Structures of depsides and benzofurans included in this study.

Following full characterisation (see Supplementary Materials) and comparison with reference databases, the compounds investigated in this study were identified as *R*-(+)-usnic acid (**1**) from *A. ochroleuca*, atranorin (**3**) from *E. cirrhatum*, decarboxythamnolic acid (**6**) and thamnolic acid (**7**) from *T. vermicularis*, and perlatolic acid (**8**) from *S. ramulosum*. In addition, the synthetic derivatives included dibenzoyl usnic acid (**2**), 2,4-bis(benzoyloxy)atranorin (**4**), and 4-*O*-methyl atranorin (**5**) (Figure 1).

This compound set was selected to encompass structural diversity within depsides and benzofuran-related scaffolds, enabling evaluation of structure–activity relationships (SAR) and their correlation with calculated electronic and thermodynamic descriptors.

### 3.2. Electronic Descriptors Correlated with Antioxidant Activity

The calculated electronic and thermodynamic parameters for the studied compounds are summarised in Table 1. Enthalpies of formation (ΔHf) ranged from 52.95 to 24.83 kJ·mol^−1^, with the lowest value observed for compound **7** and the highest for compound **5**, reflecting relative differences in thermodynamic stability. HOMO energies varied from −9.675 to −8.875 eV, with compound **7** exhibiting the lowest (most stabilised) value and compound **2** the highest. The ΔH(H-1) descriptor ranged from 0.168 to 0.655 (arbitrary units), with compound **8** showing the lowest value and compound **6** the highest. LUMO energies (LUMO_r_) ranged from −1.633 to −0.423 eV, with the most stabilised orbital associated with compound **1** and the least stabilised with compound **8**.

**Figure 2:**
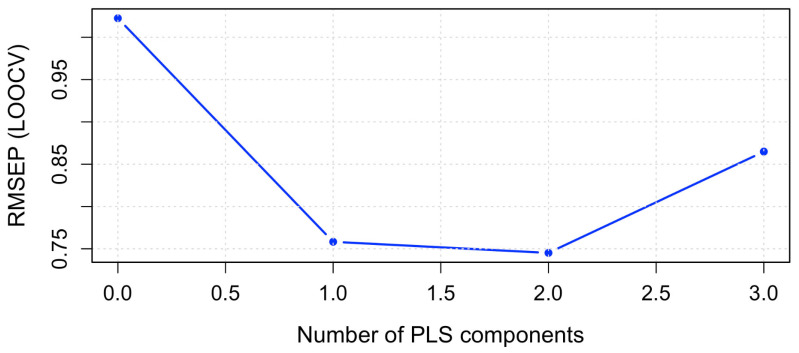
Residual variance associated with the principal components as calculated by PLS-1.

Statistical analysis using partial least squares regression (PLS-1) revealed that a single latent variable accounted for the majority of the systematic variance in the dataset (Figure 2).

In the model correlating TEAC values (dependent variable) with ΔH(H-1), LUMO_r_, and ΔHf (independent variables), compound **6** was identified as a statistical outlier based on leave-one-out cross-validation (LOOCV) residual analysis, showing the highest prediction error across all compounds. It was therefore excluded from the final model to improve robustness.

**Figure 3:**
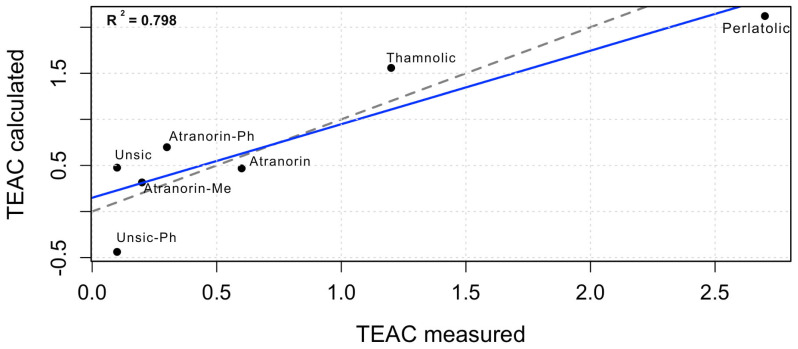
Correlation between experimental and calculated TEAC values. Compound labels correspond to those used in the graph: Usnic (*R*-(+)-usnic acid (**1**)); Usnic−Ph (dibenzoyl usnic acid (**2**)); Atranorin (**3**); Atranorin−Ph (2,4-bis(benzoyloxy)atranorin (**4**)); Atranorin−Me (4-*O*-methyl atranorin (**5**)); Thamnolic (thamnolic acid (**7**)); Perlatolic (perlatolic acid (**8**)).

Following exclusion, the refined PLS-1 model (one latent variable) explained 79.8% of the variance in TEAC values. Cross-validation confirmed improved predictive performance relative to the intercept-only model, with RMSEP decreasing from 1.023 to 0.758, supporting the predictive relevance of the selected electronic descriptors. Regression analysis indicated that ΔH(H-1) contributed most strongly to antioxidant activity, followed by LUMO_r_, whereas ΔHf exerted a comparatively smaller influence. Additional classical QSAR descriptors, including logP and polarisability, were evaluated but showed negligible contribution to the model. Overall, a moderate correlation was observed between predicted and experimental TEAC values, indicating that the computational model successfully captures the key electronic factors governing radical scavenging behaviour (Figure 3).

### 3.3. Experimental Radical Scavenging Activity

Antioxidant activity, as determined by the Trolox Equivalent Antioxidant Capacity (TEAC) assay, showed clear quantitative variation across the analysed compounds, with values ranging from 0.1 to 2.7 mmol Trolox equivalents per gram (Table 1). Compound **8** exhibited the highest radical scavenging capacity (TEAC = 2.7), followed by compounds **6** (1.4) and **7** (1.2), indicating comparatively higher activity than the remaining members of the series, whereas compounds **1** and **2** displayed the lowest activity, both showing identical TEAC values of 0.1 and therefore representing the least active compounds in the dataset. Intermediate activity was observed for compounds **3** (0.6), **4** (0.3), and **5** (0.2), confirming a clear gradation in antioxidant capacity across the series. Overall, a moderate correlation between experimental and calculated TEAC values was observed, supporting the ability of the computational model to capture key electronic trends governing radical scavenging behaviour in these compounds.

## 4. Discussion

Analysis of the studied depsides and benzofurans revealed that antioxidant activity does not directly correlate with the number of phenolic OH groups. For example, decarboxythamnolic acid (**6**) and thamnolic acid (**7**), despite exhibiting similar hydroxylation patterns, showed markedly different radical scavenging capacities. This finding indicates that no single structural feature can adequately account for antioxidant behaviour, challenging the commonly held assumption that a higher degree of hydroxylation necessarily translates into increased activity. Instead, reactivity appears to be governed by more subtle electronic effects, with ΔH(H-1), LUMO_r_ and HOMO energy emerging as the principal determinants. Collectively, these results highlight that a meaningful understanding of antioxidant activity requires an integrative framework combining structural features with electronic descriptors [35].

From a mechanistic perspective, antioxidant activity proceeds via hydrogen atom transfer, in which a radical species is neutralised through homolytic cleavage of the antioxidant O–H bond. The efficiency of this process is closely related to the bond dissociation energy (BDE), defined as the difference in enthalpy between the phenoxyl radical formed and the parent compound [36]. In the present study, BDE values were calculated for all phenolic OH groups in each compound—including *R*-(+)-usnic acid (**1**), dibenzoyl usnic acid (**2**), atranorin (**3**), 2,4-bis(benzoyloxy)atranorin (**4**), 4-*O*-methyl atranorin (**5**), decarboxythamnolic acid (**6**), thamnolic acid (**7**), and perlatolic acid (**8**)—and the lowest BDE site was selected as the representative descriptor. These structures correspond to naturally occurring depsides and benzofurans isolated from selected lichen species, as described in the Results section, alongside semisynthetic derivatives designed to evaluate the effect of esterification and methylation on both electronic properties and antioxidant activity. By restricting the chemical space to phenolic hydroxyls, the model avoids confounding contributions from aliphatic OH groups and provides a more reliable basis for interpreting electronic effects on reactivity [37].

Within this framework, ΔH(H-1) showed a clear structure–activity trend: compounds with values below 0.2, such as perlatolic acid (**8**) (ΔH(H-1) = 0.168), consistently exhibited TEAC values above 2.0. This descriptor can be interpreted as an orbital interaction term reflecting the energetic gap associated with intramolecular electron redistribution from HOMO-1 to HOMO, which facilitates spin delocalisation upon radical formation. A smaller HOMO–HOMO-1 separation therefore enhances electronic reorganisation following hydrogen abstraction, stabilising the resulting phenoxyl radical [38]. Conversely, compounds with ΔH(H-1) above 0.4, such as decarboxythamnolic acid (**6**) (ΔH(H-1) = 0.655), showed TEAC values below 0.5, indicating that increased orbital separation impedes delocalisation and diminishes antioxidant efficiency. In this context, ΔH(H-1) emerges as the most influential descriptor, followed by LUMO_r_ and ΔHf, which provide complementary information on radical stabilisation and thermodynamic contributions.

HOMO energy, as an indicator of electron-donating ability, is consistent with Koopmans’ theorem, where ionisation potential is approximated by −EHOMO. Compounds with higher HOMO energies (less negative values), such as dibenzoyl usnic acid (**2**) (HOMO = −8.875 eV), are more prone to electron donation; however, this parameter alone is insufficient to fully explain the observed activity trends. This supports the notion that descriptors involving orbital interactions, particularly ΔH(H-1), provide a more accurate description of the electronic reorganisation associated with radical scavenging, complementing HOMO-based interpretations [39].

In parallel, the LUMO energy of the corresponding phenoxyl radicals (LUMO_r_) also showed a meaningful relationship with antioxidant performance. Higher LUMO_r_ values indicate an increased capacity to accommodate additional electron density, thereby enhancing radical stabilisation and extending radical lifetime. This behaviour is exemplified by perlatolic acid (**8**), which combines a low ΔH(H-1) (0.168) with a relatively high LUMO_r_ (−0.423 eV), resulting in the highest observed activity (TEAC = 2.7). In contrast, *R*-(+)-usnic acid (**1**) and dibenzoyl usnic acid (**2**), despite possessing the theoretically favourable 2,4-dihydroxyacetophenone motif, exhibited less favourable electronic profiles (higher ΔH(H-1) and more negative LUMO_r_ values), and consequently behaved as the least active scavengers (TEAC ≈ 0.1). These findings demonstrate that the presence of structurally favourable motifs does not necessarily translate into high activity if the global electronic distribution does not support radical stabilisation [40].

The spatial arrangement of hydroxyl groups further contributes to antioxidant performance. Hydroxyl substituents located in ortho or para positions relative to carbonyl groups or conjugated systems enhance phenoxyl radical stabilisation through resonance effects and possible intramolecular interactions [41]. Accordingly, electronic orientation and conjugation exert a higher influence than the simple number of hydrogen-donating groups, explaining why compounds such as thamnolic acid (**7**) (TEAC = 1.2) may outperform more hydroxylated analogues with less favourable electronic configurations. These effects also provide a plausible explanation for the generally enhanced redox behaviour observed in lichen-derived depsides compared with their synthetic analogues, offering a chemical rationale for their traditional use in oxidative stress-related conditions.

The influence of substituents was further confirmed by the synthetic derivatives, which clearly demonstrated that electron-donating and electron-withdrawing groups significantly modify ΔH(H-1) and LUMO_r_ values, thereby altering antioxidant activity. In particular, acylation and methylation, as observed in 2,4-bis(benzoyloxy)atranorin (**4**) and 4-*O*-methyl atranorin (**5**), increased ΔH(H-1) and lowered LUMO_r_ relative to their natural precursors, resulting in reduced radical scavenging capacity [42]. This highlights that substituent–aromatic system electronic communication plays a decisive role in radical stabilisation, suggesting that optimisation strategies should prioritise electronic tuning rather than merely increasing hydroxyl content [43].

These mechanistic and electronic interpretations were further supported by multivariate analysis. Partial least squares (PLS-1) regression, after exclusion of compound **6** (decarboxythamnolic acid) identified as an outlier via LOOCV residual analysis, yielded a robust model in which a single latent variable explained 79.8% of the variance in TEAC values (R^2^Y = 0.798) with a cross-validated predictive ability of Q^2^ = 0.724, indicating acceptable robustness and limited overfitting [44]. The refined model showed improved agreement between predicted and experimental values, although the moderate predictive performance suggests that expansion of the dataset would further enhance model reliability.

Finally, classical QSAR descriptors such as logP and polarisability contributed only marginally to the model, reinforcing that antioxidant activity in this system is primarily governed by electronic rather than global physicochemical properties [45]. Overall, the results indicate that the antioxidant capacity of depsides and benzofurans is fundamentally determined by the efficiency of phenoxyl radical stabilisation and the associated electronic redistribution. In particular, the combined use of ΔH(H-1) and LUMO_r_ provides a robust quantitative framework for predicting activity and offers valuable descriptors for the rational design of new antioxidant compounds through targeted electronic optimisation of aromatic systems.

**Figure 4:**
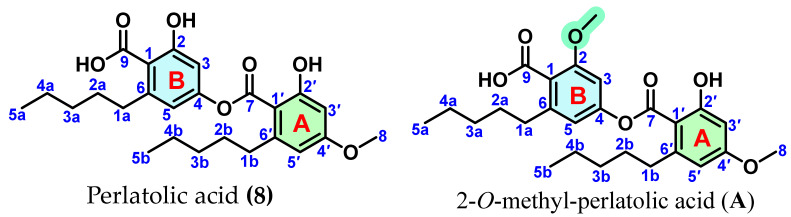
2-*O*-methyl-perlatolic acid (**A**), rationally designed derivative of perlatolic acid (**8**).

Taken together, these findings not only rationalise the observed structure–activity relationships but also provide a robust basis for the design of new antioxidant scaffolds with improved pharmacological profiles. On this basis, perlatolic acid (**8**) and *R*-(+)-usnic acid (**1**) emerges as the most informative starting points for structural optimisation within the present dataset, representing, respectively, the most active depside and a structurally privileged benzofuran framework with latent reactivity. In the case of perlatolic acid, its superior antioxidant performance (TEAC = 2.7) and favourable electronic descriptors (low ΔH(H-1) and a relatively stabilised LUMO_r_) suggest that activity is predominantly governed by a single phenolic centre, while the second hydroxyl group plays a secondary modulatory role. This asymmetry enables a targeted modification strategy; whereby selective protection of the less critical phenolic site is expected to improve pharmacokinetic properties without compromising radical scavenging capacity. Accordingly, 2-*O*-methyl-perlatolic acid (**A**) is proposed as a rational derivative, in which increased lipophilicity and metabolic stability are anticipated to enhance drug-likeness while preserving the electronic framework responsible for its high antioxidant activity (Figure 4).

**Figure 5:**
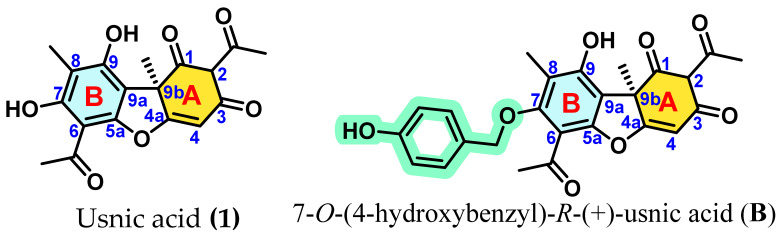
7-*O*-(4-hydroxybenzyl)usnic acid (**B**), rationally designed derivative of *R*-(+)-usnic acid (**1**).

Conversely, *R*-(+)-usnic acid, despite its low intrinsic activity (TEAC ≈ 0.1), presents a highly stable benzofuran core and a well-defined substitution pattern, making it an attractive scaffold for electronic reactivation. Its unfavourable descriptor profile (high ΔH(H-1) and a strongly stabilised LUMO_r_ in the neutral form) indicates inefficient radical stabilisation, which may be addressed through conjugation extension strategies. In this context, introduction of a para-hydroxybenzyl substituent at the most accessible phenolic position affords the 7-*O*-(4-hydroxybenzyl)usnic acid (**B**) derivative (Figure 5). This modification is expected to enhance antioxidant performance by extending π-conjugation across the benzofuran system and introducing an additional hydrogen-donating centre, thereby lowering ΔH(H-1) and improving radical stabilisation through increased electronic delocalisation.

Importantly, both proposed derivatives remain within favourable drug-like chemical space, with predicted molecular weights below 500 Da and logP values compatible with oral bioavailability, while retaining synthetic accessibility based on the methodologies validated in the present study. Collectively, these rationally designed structures exemplify the direct application of the developed QSAR model, translating electronic descriptors into actionable molecular design rules for the development of next-generation antioxidant agents with potential pharmaceutical and nutraceutical relevance.

## 5. Conclusions

The antioxidant capacity of depsides and benzofurans derived from selected lichen species was found to depend primarily on specific electronic parameters, in particular the bond dissociation energy (BDE) of the most reactive hydroxyl group, the HOMO–HOMO-1 energy gap (ΔH(H-1)), and the LUMO energy of the corresponding phenoxyl radical (LUMO_r_). Among the investigated compounds, perlatolic acid (**8**) exhibited the most favourable electronic profile and the highest antioxidant capacity (TEAC = 2.7), whereas *R*-(+)-usnic acid (**1**) and dibenzoyl usnic acid (**2**) showed the lowest activity (TEAC ≈ 0.1), highlighting the strong influence of electronic organisation on radical scavenging efficiency.

A robust quantitative structure–activity relationship was established using a PLS-1 regression model (R^2^Y = 0.798; Q^2^ = 0.724, LOOCV), confirming good predictive performance and providing a practical framework for the evaluation of antioxidant potential based on computed electronic descriptors. Within this model, the combined contribution of ΔH(H-1) and LUMO_r_ emerged as the most informative indicator of phenoxyl radical stability and overall reactivity.

Importantly, these results not only rationalise the observed structure–activity relationships but also enable the rational design of improved antioxidant scaffolds. On this basis, two optimised derivatives are proposed: 2-*O*-methyl-perlatolic acid (**A**), derived from perlatolic acid (**8**), designed to enhance lipophilicity and metabolic stability while preserving its superior electronic framework; and 7-*O*-(4-hydroxybenzyl)usnic acid (**B**), derived from *R*-(+)-usnic acid (**1**), conceived to extend π-conjugation and introduce an additional hydrogen-donating centre, thereby improving radical stabilisation and overall antioxidant performance.

These findings emphasise that antioxidant activity is governed not only by hydroxylation patterns but also by subtle electronic distribution, aromatic conjugation, and substituent effects. Although further validation using larger datasets and biological systems is required, the present approach provides a solid rational basis for the design of next-generation lichen-derived antioxidants with optimised electronic properties and potential applications in pharmaceutical and nutraceutical development.

## Figures and Tables

**Table 1:** Comparison of experimental and calculated data for depsides and benzofurans.

Compound	TEAC	ΔHf	HOMO	HOMO-1	ΔH-(H-1)	LUMOr
**1** (OH-35)	0.1	26.69	−9.392	−9.680	0.288	−1.633
**2** (OH-35)	0.1	49.24	−8.875	−9.394	0.519	−1.163
**3** (OH-24)	0.6	35.24	−9.103	−9.553	0.450	−0.858
**4** (OH-38)	0.3	28.34	−9.143	−9.526	0.383	−1.038
**5** (OH-25)	0.2	52.95	−9.077	−9.521	0.444	−0.582
**6** (OH-39)	1.4	34.11	−9.026	−9.681	0.655	−1.237
**7** (OH-39)	1.2	24.83	−9.675	−9.886	0.211	−0.873
**8** (OH-38)	2.7	26.45	−9.338	−9.506	0.168	−0.423

TEAC: Trolox Equivalent Antioxidant Capacity; ΔHf: heat of formation; HOMO: highest occupied molecular orbital; HOMO-1: next highest occupied molecular orbital; ΔH-(H-1): energy gap between HOMO and HOMO-1; LUMO_r_: lowest unoccupied molecular orbital. Compounds: **1** (OH-35), (*R*-(+)-usnic acid); **2** (OH-35), (dibenzoyl usnic acid); **3** (OH-24), (atranorin); **4** (OH-38), (2,4-bis(benzoyloxy)atranorin); **5** (OH-25), (4-*O*-methyl atranorin); **6** (OH-39), (decarboxythamnolic acid); **7** (OH-39), (thamnolic acid); **8** (OH-38), (perlatolic acid).

## Data Availability

The datasets and materials used and/or analysed during the current study are available from the corresponding author upon reasonable request.

## Supplementary Materials

The following supporting information can be downloaded at: https://www.mdpi.com/article/10.3390/antiox15050584/s1, The detailed characterisation of the compounds is provided in the Supplementary Materials.

## Abbreviations

The following abbreviations are used in this manuscript:

ABTS	2,2′-Azino-bis(3-ethylbenzothiazoline-6-sulfonic acid)
BDE	Bond Dissociation Energy
CAT	Catalase
ΔH-(H-1)	Energy difference between HOMO and HOMO-1 orbitals
GPX	Glutathione Peroxidase
HOMO	Highest Occupied Molecular Orbital
LUMO	Lowest Unoccupied Molecular Orbital
LUMO_r_	Lowest Unoccupied Molecular Orbital of the Radical
logP	Partition coefficient (measure of hydrophobicity)
PLS-1	Partial Least Squares Regression (one component)
RMS	Root Mean Square
ROS	Reactive Oxygen Species
SOD	Superoxide Dismutase
TEAC	Trolox Equivalent Antioxidant Capacity
UHF	Unrestricted Hartree–Fock
