# Supplementary material for: QSAR Analysis of Lichen Depsides and Derivatives: Electronic Descriptors as Predictors of Antioxidant Activity via PLS-1
Source: Antioxidants (Basel). 2026 May 5;15(5):584. doi: 10.3390/antiox15050584 (PMC13203360; doi:10.3390/antiox15050584)
Supplement: Supplementary file 1 [file antioxidants-15-00584-s001.zip › antioxidants-4204328-supplementary.pdf]

Supplementary Materials

QSAR Analysis of Lichen Depsides and Derivatives: Electronic Descriptors as Predictors of Antioxidant Activity via PLS-1

Patricia Mollinedo[1], José Luis Vila[1], Paola Nogales-Ascarrunz[1], and Luis Apaza Ticona[2,*]

[1] Chemistry Research Institute, Faculty of Pure and Natural Sciences, Universidad Mayor de San Andrés, Building, 2nd Floor, Laboratory 6, Calle 27, Cota Cota, University Campus, La Paz, Bolivia; pmollinedo@fcpn.edu.bo (P.M.); jlvila@fcpn.edu.bo (J.L.V.); pnogalesa@fcpn.edu.bo (P.N.-A.)

[2] Organic Chemistry Unit, Department of Chemistry in Pharmaceutical Sciences, Faculty of Pharmacy, University Complutense of Madrid, Plza. Ramón y Cajal s/n, 28040 Madrid, Spain

* Correspondence: lnapaza@ucm.es (L.A.T.)

1. Characterisation of Compounds

1.1. Compound 1

Revealed sixteen protons and eighteen carbons, consistent with the molecular formula $C_{18}H_{16}O_7$. The ^{1}H NMR spectrum displayed four well-resolved methyl singlets at δ_H 1.77 (CH_3-9b, aliphatic bridge in ring B), 2.14 (CH_3-8, aromatic in ring B), 2.67 (CH_3CO-2 in ring A), and 2.68 (CH_3CO-6 in ring A). These signals were further supported by a characteristic a vinylic (enolic) proton at δ_H 5.98 (s, H-4, bridging rings A and B) and a strongly deshielded phenolic hydroxyl at δ_H 11.10 (s, OH-9 in ring B), consistent with strong intramolecular hydrogen bonding (chelation).

In agreement with the above data, the ^{13}C NMR spectrum revealed eighteen distinct signals, comprising four methyl carbons, one methine carbon (C-4 in the bridging position), and thirteen quaternary carbons. Among these, three conjugated carbonyl carbons were particularly notable: δ_C 198.5 corresponding to the ketone at C-1 bridging rings A and B, δ_C 202.2 for the acetyl group at C-2 in ring A, and δ_C 200.8 for the acetyl group at C-6 in ring A. Additionally, the oxygenated phenolic carbons C-7 (δ_C 164.3 in ring B) and C-9 (δ_C 157.9 in ring B) appeared within the expected 150–165 ppm region, while the signal at C-9b (δ_C 59.5, quaternary sp^3) is diagnostic of the bicyclic bridge characteristic of the dibenzofuran system, corresponding to the fusion of rings A and B via C-4a and C-9b.

Based on HMBC correlations, the highly substituted dibenzofuran-type skeleton framework was clearly established. Specifically, the vinylic proton H-4 (δ_H 5.98, s) exhibited long-range correlations with C-4a (δ_C 179.8), C-9b (δ_C 59.5), and C-3 (δ_C 147.1). Additional correlations with C-3a and C-9a further support its position within the bridging vinylic system connecting rings A and B. The acetyl group CH3CO-6 (δ_H 2.68) displayed cross-peaks to C-6a (δ_C 101.9), its carbonyl CO-6 (δ_C 200.8), C-6, C-5a, and C-7, thereby establishing acetyl substitution at C-6 of ring A. Likewise, CH_3CO-2 (δ_H 2.67) correlated with C-4a (δ_C 105.7), CO-2 (δ_C 202.2), C-2, C-3, and C-1, corroborating its location at C-2 of ring A adjacent to the bridge.

Moreover, the methyl group of the bridge, CH_3-9b (δ_H 1.77), exhibited key HMBC correlations with C-9b, C-4a, C-8 (δ_C 109.7 in ring B), the central ketone C-1 (δ_C 198.5), C-9, C-9a, and C-5a, thereby defining the bicyclic skeleton connecting rings A and B. The aromatic methyl CH_3-8 (δ_H 2.14) correlated with C-8, C-9 (δ_C 157.9, phenolic in ring B), C-7 (δ_C 164.3, phenolic in ring B), C-8a, and C-9a, establishing its diagnostic position between two oxygenated phenolic carbons in ring B. Finally, the phenolic proton OH-9 (δ_H 11.10) displayed correlations with C-9, C-8, C-9a, and C-8a, corroborating the chelated phenolic arrangement that is crucial for the conformational stability of usnic acid. Supporting the structural elucidation, FABMS analysis showed a protonated molecular ion at *m/z* 345 [M+H]$^+$, definitively confirming the molecular formula $C_{18}H_{16}O_7$ for *R*-(+)-usnic acid (**1**).

1.2. Compound 2

Was synthesised via esterification with benzoyl chloride in the presence of chloroform and pyridine, yielding dibenzoyl usnic acid. The ^{1}H NMR spectrum indicated that the singlet at δ_H 10.41 corresponds to the proton of the aromatic hydroxyl group at C-9 in ring B. The aromatic protons of the two benzoyl groups (eight protons in total) are distributed between δ_H 7.00 and 8.00 ppm, reflecting the presence of two benzoyl substituents with

similar electronic environments. Simultaneously, the vinylic proton of the lactone at C-4, bridging rings A and B, appears as a singlet at δ_H 5.92, whereas the H-12a and H-12b protons of the exocyclic CH_2 at C-1, at the A/B junction, appear as doublets at δ_H 5.12 and 5.45 with a coupling constant J = 1.76 Hz. The three methyl groups are also clearly distinguished: the aromatic acetyl attached to C-6 in ring A at δ_H 2.49, the methyl on the aromatic quaternary carbon C-8 in ring B at δ_H 1.96, and the methyl on the non-aromatic quaternary carbon C-9b in the central lactone bridge at δ_H 1.74.

These data are supported by the ^{13}C NMR spectrum, which is consistent with the proposed assignments. The exocyclic carbon C-1 is observed at δ_C 201.2, while the carbon of the aromatic acetyl group at C-6 in ring A appears at δ_C 200.9. The lactone functionality is reflected in C-4a at the A/B bridge (δ_C 174.2), whereas the benzoyl-protected hydroxyl carbon at C-3 in ring A is detected at δ_C 165.4. The carbonyl carbons of the benzoyl groups are identified at δ_C 164.7 and 163.3. The aromatic carbons bearing hydroxyls at C-7 and C-9 in ring B are located at δ_C 164.2 and 157.8, respectively. The vinylic CH C-12 is assigned at δ_C 135.0, while the exocyclic CH2 appears at δ_C 110.2. The aromatic quaternary carbons C-8, C-9a, and C-6 are observed at δ_C 109.3, 104.3, and 102.1, respectively, and the quaternary carbon C-9b of the lactone system is recorded at δ_C 61.0. Finally, the quaternary carbon bearing the *O*-benzoyl group, C-2 in ring A, is detected at δ_C 114.9.

On this basis, HMBC experiments confirmed the expected long-range correlations in detail. The methyl CH_3-9b (δ_H 1.74) exhibited key correlations with C-9b (δ_C 61.0, quaternary bridge carbon), C-1 (δ_C 201.2, exocyclic carbonyl), C-4a (δ_C 174.2, lactone carbonyl), C-9a (δ_C 104.3, fused ring B), C-8a, and C-5a, confirming its position in the central lactone nucleus connecting rings A and B. CH_3-8 (δ_H 1.96) showed clear correlations with C-8 (δ_C 109.3, methyl-bearing), C-9 (δ_C 157.8, oxygenated phenolic in ring B), C-7 (δ_C 164.2, oxygenated phenolic in ring B), C-8a, and C-9a, firmly establishing its position between two phenolic sites in ring B. Likewise, the acetyl group CH_3CO-6 (δ_H 2.49) correlated with C-6 (δ_C 102.1, acetyl-bearing carbon), CO-6 (δ_C 200.9, acetyl carbonyl), C-9a (δ_C 104.3), C-6a, and C-5a, confirming precise substitution at C-6 in ring A.

The vinylic protons H-12a/b (δ_H 5.12/5.45) showed multiple correlations with C-2 (δ_C 114.9, benzoyl-bearing), C-1 (δ_C 201.2, exocyclic carbonyl), C-12 (δ_C 135.0, olefinic), C-2a, and C-3, delineating the geometry of the exocyclic double bond at the A/B junction. Meanwhile, H-4 (δ_H 5.92) exhibited long-range correlations with C-4a (δ_C 174.2, lactone), C-2 (δ_C 114.9), C-3 (δ_C 165.4, benzoyl-protected), C-9b (δ_C 61.0), and C-4b, confirming its pivotal location in the vinylic bridge of the lactone linking both rings. Similarly, the aromatic hydroxyl proton OH (δ_H 10.41) displayed specific correlations with C-9a (δ_C 104.3), C-9 (δ_C 157.8), C-8, and C-8a, verifying its location at the free phenolic site of ring B remaining after selective esterification. Supporting the structural elucidation further, FABMS analysis revealed a protonated molecular ion at m/z 569 $[M+H]^+$, consistent with the molecular formula $C_{32}H_{24}O_9$, thus confirming the identity of the compound as dibenzoyl usnic acid (**2**).

1.3. Compound 3

^{1}H NMR spectrum revealed three aromatic methyl groups at δ_H 2.11 (singlet, H-8′, three protons, C-3′ of ring B), δ_H 2.59 (singlet, H-9′, three protons, C-6′ of ring B), and δ_H 2.71 (singlet, H-9, three protons, C-6 of ring A), together with a methoxy group at δ_H 4.05 (singlet, three protons, OCH_3-7′). The aromatic protons appeared at δ_H 6.44 (singlet, H-5 of ring A) and δ_H 6.56 (singlet, H-5′ of ring B). In addition, four deshielded signals at δ_H 10.39 (formyl H-8), δ_H 11.95 (OH-2′), δ_H 12.53 (OH-2), and δ_H 12.59 (OH-4) confirmed the presence of chelating hydroxyl groups, characteristic of depsides. These deshielded hydroxyl and aldehydic signals provide evidence for intramolecular hydrogen bonding within the tetrasubstituted aromatic framework.

The ^{13}C NMR and DEPT-135 spectra revealed nineteen carbons, consistent with the complete structure of atranorin. Ring A carbons were assigned as follows: C-1 103.1, C-2 (δ_C ca. 160 ppm), C-3 108.8, C-4 161.2, C-5 113.1, and C-6 152.6. Ring B carbons included C-1′ 110.5, C-2′ (δ_C ca. 160 ppm), C-3′ 117.0, C-4′ 152.2, C-5′ 116.2, and C-6′ 140.1. Additional carbons included the methyl groups CH_3-8′, CH_3-9′, and CH_3-9, a formyl carbon C-8 (δ_C ca. 190 ppm), ester carbonyls C-7 and C-7′ at 170 and 172.4 respectively, and the methoxy carbon at δ_C 52. All DEPT-135 signals were consistent with the assignments: methyl's and methine carbons positive, quaternary carbons including carbonyls non-protonated.

HMBC correlations enabled the unambiguous assignment of all substituents and connectivity. The methyl protons H-8′ (δ_H 2.11) correlated with C-1′, C-3′, and C-5′ of ring B, establishing placement at C-3′, and showed additional long-range correlations to C-2′ and C-4′ supporting the tetrasubstituted pattern. H-9′ (δ_H 2.59)

correlated with C-5′, C-6′, and C-1′, corroborating its location at C-6′. In ring A, H-9 (δ_H 2.71) correlated with C-1, C-5, and C-6, thereby situating the methyl at C-6, with additional cross-peaks to C-2 and C-4 supporting *ortho*-methyl substitution. Aromatic proton H-5 (δ_H 6.44) correlated with C-1, C-3, C-4, and C-6, defining the tetrasubstituted pattern of ring A, while H-5′ (δ_H 6.56) correlated with C-1′, C-3′, and C-4′, confirming the ester linkage at C-4′ and its adjacency to the methyl group at C-6′.

The formyl proton H-8 (δ_H 10.39) showed a direct correlation to C-3 of ring A, and also correlated with C-2 and C-4, consistent with chelation by the hydroxyl groups OH-2 and OH-4. The methoxy protons OCH_3-7′ (δ_H 4.05) correlated with the ester carbonyl C-7′, confirming the methoxycarbonyl ester at C-1′. The chelating hydroxyls displayed characteristic HMBC cross-peaks: OH-2′ to C-1′ and C-3′, OH-2 to C-1 and C-3, and OH-4 to C-3 and C-5, consistent with depside tautomerism and hydrogen-bonded phenols.

Finally, FABMS analysis confirmed a protonated molecular ion at *m*/*z* 375 $[M+H]^+$, consistent with the molecular formula $C_{19}H_{18}O_8$, confirming the identity of the compound as atranorin (**3**).

1.4. Compound 4

^{1}H NMR spectrum revealed a methoxy group at δ_H 3.95 (s), three methyl groups at δ_H 2.05 (s, H-9′/C6′), δ_H 2.31 (s, H-8′/C3′), and δ_H 2.69 (s, H-9/C6), along with two isolated aromatic protons at δ_H 6.20 (s, H-5′) and δ_H 7.30 (s, H-5). Characteristic signals of two monosubstituted phenyl rings were observed at δH 7.55 (m, 4H, *meta*), δ_H 7.75 (m, 2H, *para*), and δ_H 8.25 (m, 4H, *ortho*). A strongly deshielded aldehydic proton appeared at δ_H 10.29 (s, H-8), and a single chelated hydroxyl at δ_H 11.95 (s, OH-2′), confirming the selective protection of the original OH groups at C2 and C4 of atranorin with benzoyl groups. The deshielded H-5 (δ_H 7.30) reflects its proximity to two *ortho*-benzoyloxy and formyl substituents.

Consistent with these observations, the ^{13}C NMR spectrum revealed twenty-seven carbons, in agreement with a di-*O*-benzoylated structure. Four methyl signals were observed (δ_C ca. 8–25 ppm: CH_3-8′ 24.2, CH_3-9′/9 (δ_H ca. 10–15 ppm), two protonated aromatic CHs (ring A/B: C-5 113.1, C-5′ 116.2), twelve quaternary aromatic carbons (including two ipso C–O benzoyl carbons (δ_C ca. 130–135 ppm), *ortho* (δ_C ca. 129 ppm), *meta*/*para* (δ_C ca. 128–133 ppm), two benzoate carbonyls at δ_C 164.8 and 165.3, the ester linkage carbonyl at C-7 (δ_C ca. 165 ppm), and the characteristic methyl ester carbonyl at δ_C 172.4 (C-7′); formyl C-8 appeared at (δ_C ca. 190–192 ppm), and the methoxy group δ_C 52 ppm completes the set for $C_{27}H_{22}O_{10}$.

HMBC correlations allowed the complete and unequivocal structural assignment. The aromatic proton H-5 (δ_H 7.30, s) displayed long-range correlations with C-1 (δ_C 103.1), C-3 (δ_C 109.7), C-4 (δ_C 160.2), and the formyl carbonyl C-8 (δ_C 192.5), fully characterising the tetrasubstituted pattern of ring A: C1–ester (C7), C2–OCOPh, C3–CHO, C4–OCOPh, and C6–CH_3. The methyl H-9 (δ_H 2.69, s) correlated with C-1 (δ_C 103.1), C-5 (δ_C 113.1), and C-6 (δ_C 152.6), confirming its location at C6 adjacent to the ester linkage C7–O–C4′, with additional cross-peaks to C-7 carbonyl supporting the depside connectivity.

In ring B, H-8′ (δ_H 2.31, s) showed correlations with C-1′ (δ_C ca. 110 ppm), C-3′ (δ_C 123.3), and C-5′ (δ_C 116.2), establishing the methyl at C3′, whereas H-9′ (δ_H 2.05, s) correlated with C-5′ (116.2), C-6′ (δ_C ca. 140 ppm), and C-1′ (δ_C ca. 110 ppm), confirming its position at C6′. The proton H-5′ (δ_C 6.20, s) correlated with C-1′ (δ_C ca. 110 ppm), C-3′ (δ_C 123.3), C-4′ (δ_C 152.2), and C-6′ (δ_C ca. 140 ppm), delineating the complete substitution pattern of ring B: C1′–C7′(=O)OCH_3, C2′–OH, C3′–CH_3, C4′–O–C7, C5′–H, and C6′–CH_3.

The methoxy group at δ_H 3.95 (s, 3H) correlated directly with C-7′ (δ_C 172.4), verifying the methoxycarbonyl ester; additional HMBC to C-1′ reinforces attachment. The aldehydic proton H-8 (δ_H 10.29, s) correlated with C-3 (δ_C 109.7), confirming the formyl group's location at position 3 of ring A, with three-bond correlations to C-2 (δ_C ca. 160 ppm) and C-4 (160.2), influenced by benzoyloxy deshielding. Finally, the hydroxyl OH-2′ (δ_H 11.95, s) correlated with C-1′ (δ_C ca. 110 ppm), C-2′ (δ_C ca. 160 ppm), and C-3′ (δ_C 123.3), confirming its free position at C2′; chelation is evident from the downfield shift and HMBC to the ester C-7′ indirectly. The benzoyl protons display standard HMBC correlations: *ortho* protons to ipso carbons and carbonyls (164.8/165.3), distinguishing the two non-equivalent units at C2/C4 due to asymmetry.

FABMS analysis revealed a protonated molecular ion at *m*/*z* 503 $[M+H]^+$ (calculated 502 Da for $C_{27}H_{22}O_{10}$), fully consistent with the molecular formula, confirming the identity of 2,4-bis(benzoyloxy)atranorin (**4**).

1.5. Compound 5

^{1}H NMR spectrum revealed three characteristic methyl groups at δ_H 2.11 (s, H-8′/C3′), δ_H 2.53 (s, H-9/C6), and δ_H 2.55 (s, H-9′/C6′), accompanied by two well-resolved methoxy groups at δ_H 3.96 (s, OCH_3-7′) and δ_H 3.98 (s, OCH_3-4). Two isolated aromatic protons appeared at δ_H 6.32 (s, H-5′ of ring B) and δ_H 6.59 (s, H-5 of ring A), along with a strongly deshielded aldehydic proton at δ_H 10.30 (s, H-8). The chelating hydroxyl signals at δ_H 12.58 (OH-2) and δ_H 11.92 (OH-2′) confirmed the presence of two intact hydroxyl groups. The main structural modification involves the conversion of the original OH-4 of atranorin into a methoxy group, with slight up field shifts in methyl's (2.53 and 2.55 versus 2.59 and 2.71 in atranorin) reflecting electronic changes from the methoxy substitution at C4.

Consistent with these observations, the ^{13}C NMR spectrum revealed twenty carbons, in agreement with the formula derived from atranorin ($C_{19}H_{18}O_8$ plus CH_2 giving $C_{20}H_{20}O_8$). Two ester carbonyls were identified C-7 (δ_C ca. 170 ppm), C-7′ 172.8, along with two methoxy groups OCH_3-4 (δ_C ca. 56 ppm), OCH_3-7′ (δ_C ca. 52 ppm), three aromatic methyl's CH_3-8′ (δ_C ca. 9 ppm), CH_3-9 and CH_3-9′ (δ_C ca. 10–12 ppm), a formyl group C-8 (δ_C ca. 191 ppm), and fourteen aromatic carbons arranged in two tetrasubstituted rings. Key chemical shifts include C-4 at 165.2 (quaternary OCH_3), C-6 150.1 (ring A), and C-6′ 140.0 (ring B), reflecting the methylation effect.

HMBC correlations enabled the complete and unequivocal structural assignment. The methyl H-8′ (δ_H 2.11, s) showed long-range correlations with C-1′ (δ_C 110.2), C-3′ (δ_C 117.5), and C-5′ (δ_C 116.8), thereby establishing its position at C-3′ of ring B, with additional correlations to C-2′ and C-4′ confirming its meta relationship. The methyl H-9 (δ_H 2.53, s) correlated with C-1 (δ_C 114.2), C-5 (δ_C 104.2), and C-6 (δ_C 150.1), corroborating its position at C-6 of ring A adjacent to the ester carbonyl, whereas H-9′ (δ_H 2.55, s) correlated with C-1′, C-5′, and C-6′, confirming its location at C-6′. Both methyl groups exhibited three-bond correlations with their respective ester carbonyls.

The aromatic protons H-5 (δ_H 6.59, s) and H-5′ (δ_H 6.32, s) exhibited key correlations defining the substitution patterns of rings A and B. In ring A, H-5 correlated with C-1 (δ_C 114.2), C-3 (δ_C 109.2), C-4 (δ_C 165.2), and C-6 (δ_C 150.1), confirming the tetrasubstituted pattern: C1 connected to C7(=O)–O–C4′, C2 bearing OH, C3 connected to CHO, C4 bearing OCH_3, C5 as H, and C6 as CH_3, with additional HMBC correlations to the formyl carbon C-8 (δ_C ca. 191 ppm) and ester carbonyl C-7 (δ_C ca. 170 ppm). In ring B, H-5′ correlated with C-1′ (δ_C 110.2), C-3′ (δ_C 117.5), and C-4′ (δ_C 153.6), delineating the substitution pattern: C1′ connected to C7′(=O)OCH_3, C2′ bearing OH, C3′ connected to CH_3, C4′ connected to O–C7, C5′ as H, and C6′ as CH_3, with additional correlations to C-7′ (δ_C 172.8).

The aldehydic proton H-8 (δ_H 10.30, s) correlated directly with C-3 (δ_C 109.2), confirming the formyl group at position 3 of ring A, and showed three-bond HMBC correlations to C-2 (δ_C ca. 162 ppm, OH-chelating) and C-4 (165.2, OCH_3). The methoxy groups were specifically assigned: OCH_3-7′ (δ_H 3.96, s) correlated with C-7′ (δ_C 172.8), confirming the methoxycarbonyl ester at C1′, while OCH_3-4 (δ_H 3.98, s) correlated with C-4 (δ_C 165.2), establishing the key modification relative to atranorin, i.e., replacement of OH-4 with a methoxy group. Mutual NOE between OCH_3-4 and H-5 confirmed their peri relationship. The hydroxyls OH-2 (δ_H 12.58, s) and OH-2′ (δ_H 11.92, s) displayed typical intramolecular chelation correlations with adjacent carbons—OH-2 correlating with C-1 (114.2) and C-3 (109.2), OH-2′ correlating with C-1′ (110.2) and C-3′ (117.5)—confirming their structural integrity and hydrogen-bonding to the formyl and ester groups.

Supporting the structural elucidation further, FABMS analysis revealed a protonated molecular ion at *m*/*z* 389 $[M+H]^+$ (calculated 388 Da for $C_{20}H_{20}O_8$), consistent with the molecular formula, confirming the identity of the compound as 4-*O*-methyl atranorin (**5**).

1.6. Compound 6

^{1}H NMR spectrum revealed the presence of two distinct aromatic methyl groups, at δ_H 2.28 (singlet, three protons, H-3′) and δ_H 2.50 (singlet, three protons, H-8), a characteristic methoxy group at δ_H 3.90 (singlet, three protons, OCH_3-4), and two isolated aromatic protons at δ_H 6.38 (singlet, one proton, H-5′) and δ_H 6.65 (singlet, one proton, H-5), assigned to rings B and A, respectively. In addition, a strongly deshielded aldehydic proton appeared at δ_H 10.23 (singlet, one proton, H-8′), accompanied by an intramolecularly hydrogen-bonded hydroxyl signal at δ_H 12.45 (singlet, one proton, OH-2), typical of a hydroxyl *ortho* to an ester carbonyl, and a

free phenolic hydroxyl at δ_H 8.95 (singlet, one proton, OH-2′). The deshielded OH-2 confirms chelation with the ester carbonyl at C-7, whereas the up field OH-2′ indicates the absence of an ortho carboxyl group, supporting a decarboxylated structure relative to thamnolic acid.

Consistent with these observations, the ^{13}C NMR and DEPT-135 spectra revealed eighteen carbons, including an ester carbonyl at δ_C 169.5 (C-7), a formyl carbonyl at δ_C 194.9 (C-8′), a methoxy group at δ_C 52.1 (OCH_3-4), two aromatic methyl's at δ_C 17.9 (C-7′) and δ_C 25.4 (C-8), three protonated aromatic carbons, and eleven quaternary aromatic carbons distributed over the two tetrasubstituted phenolic rings. The DEPT-135 experiment confirmed three aromatic methines (positive signals), two methyl carbons (positive signals), one aldehydic carbon (positive signal), and eleven quaternary carbons, verifying a total of eighteen carbons, supporting the proposed decarboxylated structure.

HMBC correlations provided conclusive evidence for the placement of substituents and the overall connectivity of the molecule. In ring A, the methyl protons H-8 (δ_H 2.50) correlated with C-1 (δ_C 112.4), C-5 (δ_C 106.3), and C-6 (δ_C 145.1), corroborating its position at C-6 adjacent to the ester carbonyl linking to C-4′ of ring B. In ring B, the methyl protons H-3′ (δ_H 2.28) correlated with C-1′ (δ_C 104.2), C-3′ (δ_C 109.8), and C-5′, thereby establishing its position at C-3′. The aromatic proton H-5 in ring A (δ_H 6.65) showed correlations with C-1, C-3 (δ_C 102.1), and C-4 (δ_C 165.8), fully characterising the tetrasubstituted pattern of ring A with C1–C7(=O)–O–C4′, C4–OCH_3, and C6–CH_3 (C-8). In ring B, H-5′ (δ_H 6.38) correlated with C-1′, C-3′, C-4′ (δ_C 154.0), and the aldehydic C-8′, delineating the substitution pattern as C-1′–CH_3 (C-7′), C-3′–CH_3, C-8′–CHO, C-4′–O–C7, and C-2′–free OH, with no C-9′ carboxyl present. Long-range correlations of H-5 to oxygenated C-2 and *meta* C-6, and H-5′ spanning the tetrasubstituted ring B, fully support these assignments.

The aldehydic proton H-8′ (δ_H 10.23) displayed correlations to C-3′, confirming its placement at C-3′ of ring B. The methoxy group OCH_3-4 (δ_H 3.90) correlated directly with C-4 (δ_C 165.8), verifying the *O*-methylation. The hydroxyl OH-2 (δ_H 12.45) exhibited a deshielded shift indicative of intramolecular chelation with C-7, whereas OH-2′ (δ_H 8.95) was consistent with a free phenol. These NMR observations, together with the absence of the carboxylic carbonyl signal near 170 ppm and the chemical shift of C-2′ (δ_C 162.3), confirm decarboxylation and the presence of a free phenolic carbon at C-2′.

Finally, FABMS analysis confirmed a protonated molecular ion at *m*/*z* 377 $[M+H]^+$, consistent with the molecular formula $C_{18}H_{16}O_9$, which is 44 mass units lower than thamnolic acid ($C_{19}H_{16}O_{11}$), corresponding to the loss of CO_2, indicating that the compound corresponds to decarboxythamnolic acid (**6**).

1.7. Compound 7

^{1}H NMR spectrum revealed simplified aromatic resonances, reflecting the high degree of substitution on both aromatic rings, designated as rings A and B. In ring A, H-5 resonated as a singlet at δ_H 6.15, attributable to the symmetrical environment created by the methoxy group at C-4 and the methyl substituent at C-6 (C-8), while in ring B, H-5′ was observed as a singlet at δ_H 6.13, positioned between the formyl group at C-6′ (C-8′) and the hydroxyl at C-5′. The singlet nature of these signals is consistent with the absence of vicinal protons, as expected in heavily substituted orcinol-derived systems. Three methyl signals were also observed: δ_H 3.86 corresponding to OCH_3-4 at C-4, δ_H 2.50 for CH_3-8 attached to C-6 of ring A, and δ_H 2.45 for CH_3-7′ at C-3′ of ring B. Additionally, the aldehyde proton H-8′ appeared deshielded at δ_H 10.40, characteristic of phenolic aldehydes, with the integration of each methyl group corresponding to three protons and the aldehyde proton to one proton, consistent with the proposed structure in which C-10 is OCH_3–C4, C-8 is CH_3–C6, C-7′ is CH_3–C3′, and C-8′ is CHO–C6′.

These assignments were supported by the ^{13}C NMR and DEPT-135 spectra, which confirmed the presence of nineteen carbons, including two aromatic methine carbons observed as singlets (C-5, δ_C 106.5 and C-5′, δ_C 129.9). Key carbonyl carbons were also identified: the formyl carbon at C-8′ (δ_C 194.9), the ester carbon at C-7 (δ_C 172.5) linking C-1 of ring A to O–C4′ of ring B, and the carboxylic acid carbons at C-9 (δ_C 164.5) and C-2′ (δ_C 166.8). Oxygenated quaternary carbons were assigned to C-4 (δ_C 161.7) and C-2 (δ_C 161.0) in ring A, and C-4′ (δ_C 157.3), C-6 (δ_C 145.4) and C-6′ (δ_C 143.9) in rings A and B, appearing downfield as expected for oxygenated sp^2 carbons. The DEPT-135 spectrum confirmed the two aromatic methines (C-5 and C-5′) as positive signals, the oxygenated quaternary carbons and formyl aldehyde as non-protonated, and the methyl carbons were clearly

assigned as C-8 (δ_C 22.3), C-7′ (δ_C 16.4), and OCH_3-4 (δ_C 55.6), consistent with the structural framework, in which carboxylic acids decorate C-3 of ring A and C-2′ of ring B, while the ester linkage connects C-1–C-7–O–C4′.

Subsequently, HMBC correlations precisely delineated the depsidone connectivity. In ring A, the methyl protons H-8 (δ_H 2.50) exhibited correlations with C-1 (δ_C 112.5), C-5 (δ_C 106.5), and C-6 (δ_C 145.4), confirming the methyl substitution at C-6, while H-5 (δ_H 6.15) correlated with C-1, C-3 (δ_C 105.5), and C-8, supporting the resorcinol core. Three-bond correlations extended across the quaternary C-6, and two-bond correlations connected H-5 to C-1 and C-3 *ortho* carbons. In ring B, the methyl protons H-7′ (δ_H 2.45) correlated with C-1′ (δ_C 110.2), C-5′ (δ_C 129.9), and C-6′ (δ_C 143.9), unambiguously placing the methyl group at C-3′, with an additional one-bond correlation to C-3′ (δ_C 109.5). The aldehydic proton H-8′ (δ_H 10.40) showed two- and three-bond correlations to C-4′ (δ_C 157.3), C-5′, and C-3′, validating the formyl group at C-6′, while the methoxy protons (OCH_3-4, δ_H 3.86) were directly assigned to C-4 (δ_C 161.7) via a one-bond correlation, confirming its placement in ring A.

Finally, mass spectrometric analysis supported the molecular formula $C_{19}H_{16}O_{11}$, with a protonated molecular ion at *m/z* 421 $[M+H]^+$, consistent with thamnolic acid (**7**).

1.8. Compound 8

^1H NMR spectrum revealed the characteristic aromatic resonances of two distinct phenolic rings, designated as rings A and B. In ring B, H-5′ appeared at δ_H 6.72 (d, *J* = 1.7 Hz) and H-3′ at δ_H 6.63 (d, *J* = 1.7 Hz), displaying *meta* couplings consistent with substitution at C-1′ (δ_C 109.4), C-2′ (OH), C-4′ (OCH_3, δ_C 155.5), and C-6′ (pentyl chain 1b–5b, δ_C 150.5). The small coupling constant of approximately 1.7 Hz is characteristic of *meta* coupling and supports a 1′,3′,4′,5′,6′ substitution pattern in ring B, placing H-5′ *ortho* to the methoxy group at C-4′ and *meta* to H-3′. In parallel, ring A displayed signal overlap and broadening for H-3 and H-5 at δ_H 6.39 (broad singlet), consistent with a resorcinol-type system. This assignment is supported by the corresponding carbon signals at C-3 (δ_C 99.4) and C-5 (δ_C 111.9), together with the strongly deshielded C-2 (δ_C 166.5), indicative of a phenolic hydroxyl group. The substitution pattern in ring A, defined by COOH-9 at C-1 (δ_C 104.0), an ester functionality at C-4 (δ_C 165.3), and a pentyl substituent at C-6 (δ_C 148.9), establishes a 1,3,5-trisubstituted aromatic system, which accounts for the observed signal overlap and broadening through a combination of symmetry, restricted rotation, and hydrogen-bonding effects.

This structural framework is further supported by the aliphatic region, where two n-pentyl chains were clearly identified and unambiguously assigned to rings A and B. The left-hand chain (1a–5a) is attached to C-6 of ring A, while the right-hand chain (1b–5b) is attached to C-6′ of ring B. The benzylic protons H-1a and H-1b both appeared at δ_H 2.96 as triplets with a coupling constant of approximately 7 Hz, consistent with their proximity to the aromatic systems. These were followed by multiplets corresponding to H-2a and H-2b at δ_H 2.65, H-3a and H-4a at δ_H 2.40, and H-3b and H-4b at δ_H 2.25, with the terminal methyl groups H-5a and H-5b appearing at δ_H 0.93 and 0.87, respectively. The overall integration pattern, comprising approximately four benzylic protons, four beta methylene protons, four gamma and delta methylene protons differentiated by the distinct electronic environments of rings A and B, and six terminal methyl protons, is supporting two aromatic *n*-pentyl substituents. The presence of a methoxy group on ring B is further confirmed by the singlet at δ_H 3.85 assigned to OCH_3-4′.

The ^{13}C NMR spectrum provides complementary evidence supporting this substitution pattern and the connectivity between both rings. In particular, the ester carbonyl carbon C-7 at δ_C 175.8 establishes the linkage between C-4 of ring A and C-1′ of ring B, while the carboxylic acid carbon C-9 at δ_C 169.8 is located at C-1 of ring A. Oxygenated aromatic carbons were consistently assigned to C-2 and C-4 in ring A, and to C-4′ and C-6′ in ring B, together with C-6 in ring A, all appearing in the expected deshielded region. Protonated aromatic carbons were observed at C-1, C-3, and C-5 in ring A, and at C-1′ and C-5′ in ring B, supporting the proposed substitution patterns. The relatively downfield position of C-5′ at δ_C 116.7 reflects the combined *ortho* and *meta* electronic influence of the methoxy substituent, whereas the up field shift of C-3 at δ_C 99.4 is characteristic of a resorcinol framework in ring A.

Further confirmation is provided by the DEPT-135 experiment, which clearly distinguishes the aliphatic carbon types. Eight methylene carbons were observed as negative signals and assigned sequentially from the benzylic positions C-1a (δ_C 37.7) and C-1b (δ_C 29.6), through C-2a and C-2b (δ_C 32.5 and 32.4), C-3a and C-3b (δ_C

32.3 and 31.8), and C-4a and C-4b (δ_C 23.0 and 22.9), to the terminal methyl carbons C-5a and C-5b at δ_H ca. 14 ppm, which appeared as positive signals. This distribution of chemical shifts follows the expected trend for aliphatic chains attached to aromatic systems, with progressive shielding observed along the chain as the distance from the aromatic ring increases.

Crucially, the HMBC experiment establishes the long-range connectivity required to confirm the depsidone framework. The benzylic protons H-1a showed correlations with C-6 and C-1 of ring A, as well as a three-bond correlation with C-5, thereby anchoring the pentyl chain at C-6 of ring A. Similarly, H-1b showed correlations with C-6′ of ring B and a three-bond correlation with C-5′, unambiguously placing the second pentyl chain at C-6′ of ring B. The aromatic protons H-5′ and H-3′ exhibited correlations with multiple carbons within ring B, including C-1′, C-3′, C-4′, and C-6′, consistent with the proposed substitution pattern, while the methoxy protons showed a direct one-bond correlation with C-4′, confirming its assignment. In ring A, H-3 and H-5 displayed correlations with C-1, C-2, and C-4, and additionally showed a three-bond correlation with the ester carbonyl C-7, thereby reinforcing the connectivity between both aromatic rings through the ester linkage.

Finally, mass spectrometric analysis provides independent confirmation of the proposed structure. The FABMS spectrum exhibited a protonated molecular ion at *m*/*z* 445 corresponding to the molecular formula $C_{25}H_{32}O_7$, while the observed fragment ions are fully consistent with cleavage of the ester linkage.
